# Notch Signaling Suppresses Melanoma Tumor Development in BRAF/Pten Mice

**DOI:** 10.3390/cancers15020519

**Published:** 2023-01-14

**Authors:** Dareen Mikheil, Kirthana Prabhakar, Tun Lee Ng, Sireesh Teertam, B. Jack Longley, Michael A. Newton, Vijayasaradhi Setaluri

**Affiliations:** 1 Department of Dermatology, School of Medicine and Public Health, University of Wisconsin-Madison, Madison, WI 53705, USA; 2 Department of Biostatistics and Medical Informatics, School of Medicine and Public Health, University of Wisconsin-Madison, Madison, WI 53706, USA; 3 William S. Middleton Memorial Veterans’ Hospital, Madison, WI 53705, USA

**Keywords:** Notch signaling, melanoma, melanoma tumor development, melanoma TMA, melanoma mouse model

## Abstract

**Simple Summary:**

It is now well recognized that alterations in both oncogenic and tumor suppressor genes are required for cutaneous melanoma tumorigenesis. Although Notch signaling has been implicated in many aspects of melanoma biology, the exact role *Notch* genes play in melanomagenesis is not completely understood. Employing *BRAF^CA^*/*Pten^-/-^* mice, a widely used genetic mouse model of melanoma, we set out to investigate the role of *Notch* genes in melanomagenesis. Furthermore, using a quantitative immunohistochemical analysis of a clinically annotated human primary melanoma tissue microarray, we asked whether the amount of NOTCH proteins present in the tumor correlate with the aggressiveness of the tumor as measured by the development of metastatic disease in patients. Our data show that *Notch1* and *Notch2* genes do not function as tumor suppressor genes, unlike *Pten*, i. e., the loss of *Notch1* or *Notch 2* or both fail to induce melanoma in *BRAF^CA^* mice. However, the loss of *Notch1* or *Notch2* accelerated melanomagenesis. Once the melanoma tumor is initiated, the amount of NOTCH protein expressed in human melanoma does not appear to reliably predict melanoma aggressiveness. Therefore, identifying and targeting signaling pathways regulated by Notch may be a useful strategy for melanoma prevention.

**Abstract:**

Both oncogenic and tumor suppressor roles have been assigned to Notch signaling in melanoma. In clinical trials, Notch inhibitors proved to be ineffective for melanoma treatment. Notch signaling has also been implicated in melanoma transdifferentiation, a prognostic feature in primary melanoma. In this study, we investigated the role of Notch signaling in melanoma tumor development and growth using the genetic model of mouse melanoma by crossing *BRAF^CA/+^/Pten^+/+^/Tyr-Cre*ER+ (B) and *BRAF^CA/+^/Pten^-/-^/Tyr-Cre*ER + (BP) mice with *Notch1* or *Notch2* floxed allele mice. The topical application of tamoxifen induced tumors in BP mice but not in B mice with or without the deletion of either *Notch1* or *Notch2*. These data show that the loss of either *Notch1* nor *Notch2* can substitute the tumor suppressor function of Pten in BRAFV600E-induced melanomagenesis. However, in *Pten*-null background, the loss of either *Notch1* or *Notch2* appeared to accelerate BRAFV600E-induced tumor development, suggesting a tumor suppressor role for Notch1 and Notch2 in BRAFV600E/Pten-null driven melanomagenesis. Quantitative immunochemical analysis of a human cutaneous melanoma tissue microarray that consists of >100 primary tumors with complete clinical history showed a weak to moderate correlation between NOTCH protein levels and clinical and pathological parameters. Our data show that Notch signaling is involved during melanomagenesis and suggest that the identification of genes and signaling pathways downstream of Notch could help devise strategies for melanoma prevention.

## 1. Introduction

Melanoma is among the most rapidly increasing cancers in the U.S [1]. Unlike gender-specific cancers such as breast cancer in females and prostate cancer in males, melanoma affects both males and females. Melanoma is the least common but the most aggressive form of skin cancer. The early diagnosis and surgical removal of primary melanomas is highly effective, with five-year survival rates of 99%. However, once metastasized to distant organs, the five-year survival rate in melanoma patients drops to 30% [2,3]. Although current treatments for metastatic melanoma are effective in the short-term, often the clinical response is not durable. Therefore, there is a need for early diagnosis and the reliable and accurate prediction of the aggressive behavior of primary melanoma to better manage patients diagnosed with it. 

Notch signaling has been implicated in melanoma in different studies as both an oncogenic and tumor suppressor, whether in tumor cells [4,5,6] or endothelial cells of the different organs during metastatic progression [7]. In vivo studies implicated an oncogenic role for Notch signaling in melanoma tumor growth, progression, metastasis and immunosuppression [4,8,9,10,11,12,13]. On the other hand, a mouse co-graft experiment revealed a tumor suppressor function for Notch in MAFs (Melanoma Associated Fibroblasts) [14]. In addition, Notch 1 activation was implicated in tumor angiogenesis, as *casp11* deficiency reduced angiogenesis in melanoma xenografts [15]. In NRAS wildtype melanoma, NOTCH4 mutation was suggested as a biomarker that predicts good immune response [16]. In metastatic melanoma cell lines, Skarmoutsou et. al. found that FOXP3 upregulation mediated by Notch1 signaling could potentially serve as a marker for tumor aggressiveness and metastasis, and that NOTCH1 inhibition causes the significant reduction of melanoma cell proliferation and viability [17]. Notch signaling activation causes the transcriptional upregulation of multiple oncogenes, including c-MYC, PCNA, and CDK4, and leads to melanoma progression [18].

Nevertheless, the role of Notch signaling in the early stages of melanocyte transformation and melanoma tumor formation as well as the prognostic value for Notch in primary melanoma patients are still not fully understood.

In previous studies, we showed that Notch signaling plays a role in neural-type differentiation, which better predicts the survival of patients diagnosed with primary melanoma [19]. However, it is not clear whether Notch signaling has a role in melanogenesis or whether the expression of Notch signaling proteins have a prognostic value for melanoma tumor progression. 

Here, we investigated the role of Notch signaling in melanoma tumor formation in vivo: the *BRAF^CA/+^/Pten^-/-^/Tyr-Cre*ER+ (BP) melanoma mouse model by conditional deletion of either *Notch1* or *Notch 2*. Our data show that the absence of *Notch1* or *Notch2* tends to accelerate melanoma tumor development and growth. Additionally, the loss of neither Notch1 nor Notch2 was able to substitute for Pten loss or cooperate with BRAFV600E mutation in melanomagenesis. We also used the quantitative immunochemical analysis of an in-house generated melanoma tissue microarray (TMA) to evaluate the expression of Notch signaling proteins as well as the neuronal differentiation marker microtubule-associated protein 2 (MAP2) to understand the correlation between Notch signaling and/or MAP2 and melanoma progression. Our data show that NOTCH1 expression tends to be associated with less aggressive melanoma and better survival. 

## 2. Materials and Methods

### 2.1. Generation of Notch1- and Notch2-Null Mice

B6.Cg-Tg(Tyr-cre/ERT2)13Bos *Braf^tm1Mmcm^ Pten^tm1Hwu^*/BosJ mice (Strain 013590) were obtained from the Jackson Lab (Bar Harbor, ME) and were crossed with either *Notch1^tm2Rko^*/GridJ or *Notch2^tm3Grid^*/J (generously provided by Dr. Tim King, UW-Madison). The genotyping of the resulting mice were performed by Transnetyx. A one-time topical application of 4-HT is sufficient to induce melanoma tumors on the shaved flank skin of these mice in 3–4 weeks [20]. Accordingly, 2µL of 5 mM 4-HT was applied once to four different spots on the shaved mice backs. Animals were checked regularly for tumor formation at the four spots of application. Tumors were measured (using a caliper) every other weekday until at least one of the four tumors on each mouse reached a measurement of 10 mm in any direction. The mice were euthanized using a CO_2_ chamber and tumors were collected for further immunohistochemical and western blotting analyses. All animal experiments were approved by the UW Institutional Animal Care and Use Committee.

### 2.2. Immunohistochemistry

Immediately after euthanizing the animals, mouse skin tumors were excised and fixed in 10% formalin and stored at 4 °C in 70% ethanol. The tumors were then submitted to the UWCCC Experimental Pathology Laboratory for paraffin embedding and sectioning. Slides with tumor sections were placed in an 80 °C oven for 20 min and were then deparaffinized in xylene (5 min × 3) and hydrated through graded alcohols and finally in deionized water. Antigen retrieval was done in pH6.0 citrate solution (10 mM citric acid, 0.05% tween 20) for 3 min in a Biocare decloaker (Biocare, Concord, CA, USA). Endogenous peroxidase was blocked with 0.3% H_2_O_2_ in PBS for 20 min at room temperature. After rinsing with PBS, 10% goat serum (Sigma, St. Louis, MO, USA) in PBS was used to block nonspecific binding (1 h at room temperature). Slides were then incubated overnight at 4 °C with the primary antibody in PBS with 1% goat serum at [1:200 for anti-Ki67 (Cell Signaling#12202); 1:100 for anti-Notch1 (Millipore# 07-1232); 1:100 for ant-Notch2 (Millipore#07-1234); and 1:100 for ant-S100A4 (Cell Signaling#13018)]. After rinsing with PBS, the slides were incubated with Signal Stain^®^ (Danvers, MA, USA) Boost IHC Detection Reagent (Cell Signaling) for 30 min at room temperature. Following PBS rinse, slides were developed with ImmPACT Nova Red (Vector Laboratories, Inc., Burlingame, CA, USA) for one minute and then rinsed with H_2_O. Slides were then counter stained with Mayer’s hematoxylin (Sigma, St. Louis, MO, USA) for one minute and rinsed with tap H_2_O for 10 min followed by a DH2O rinse. Slides were then dehydrated through graded alcohols to xylene and mounted.

### 2.3. Immunohistochemical Analysis of TMA

A melanoma tissue microarray was constructed in-house that consisted of 12 nevi and 168 cutaneous primary melanoma tumors annotated with clinical information including patient age, sex, recurrence of the tumor, and Breslow thickness [19,21]. This study was approved by the Institutional Review Board of the University of Wisconsin. An immunohistochemical analysis was performed on serial sections of a TMA and multispectral images were collected by a Vectra slide scanner (Caliper Life Sciences, Hopkinton, MA, USA) at the Translational Initiatives in Pathology laboratory of the UW Comprehensive Cancer Center. Automated image analysis was performed using InForm software (Akoya Biosciences, Inc., Marlborough, MA, USA). By employing the unique spectral properties for differently colored chromogens, a multispectral library was made by staining individual test melanoma sections with each chromogen to be used for the multispectral imaging. Using this library, signals from multicolored slides stained for multiple markers were separated. We used the InForm function for pattern recognition to identify staining patterns, i.e., tumor vs. normal tissue vs. non-specific artifacts and nucleus vs. cytosol.

### 2.4. Antibodies

Anti-S100 (melanoma tumor marker) (Roche Ventana #790-2914), anti-Ki67 (proliferation marker) (Abcam #ab16667), anti-NOTCH1 (Millipore Sigma 07-1231), anti-NICD (Cell signaling #4147), anti-HES1 (Cell signaling #11988), anti-MAP2 (ThermoFisherSci #PA5-24589, Waltham, MA, USA) and anti-NOTCH2 (Sigma #HPA048743) antibodies were utilized. 

### 2.5. Multiplex Chromogenic

Slides were deparaffinized in xylene and rehydrated through graded alcohols to distilled H_2_O, followed by TBS with Tween-20 (TBST). All reagents used (with catalog numbers in parentheses) for staining unless otherwise specified were from Biocare Medical, Pacheco, CA, USA. Peroxidazed 1 (PX968) was applied for 5 min to block endogenous peroxidase. Slides were then rinsed with distilled H_2_O and placed in a Coplin jar with a Borg Decloaker buffer (BD1000) for standardized heat induced epitope retrieval. Next, slides were cooled down and rinsed with DH_2_O, followed by TBS and then TBST. The tissue was delineated with a hydrophobic barrier pen and rinsed with PBS with Tween (PBST). A Background Punisher (BP974) protein block was applied to the slides for 7 min at room temperature. The block was removed from the slides and 100–125μL of Ki-67 antibody (Abcam #ab16667, 1:100 dilution) (for all slides except NICD slide, NICD antibody, Cell signaling #4147, 1:100 dilution) diluted with Da Vinci Green Diluent (PD900) was applied and the slides were placed in a humidity chamber for 1 h at room temperature. After the incubation period, the slides were thoroughly rinsed with TBS and then with TBST. After the buffer was removed, Mach 2 Rabbit HRP-Polymer (RHRP520) was applied and the slides were returned to the humidity chamber for 30 min at room temperature. After the incubation period, the slides were thoroughly rinsed with TBS, then with TBST. The buffer was removed and Betazoid DAB Chromogen (BDB2004) was applied to the slides for 3–4 min. The slides were thoroughly rinsed with distilled H_2_O, treated with Denaturing Solution (DNS001; 1 part A + 3 parts B) for 2 min, rinsed with distilled H_2_O and then finally with TBST. The block was removed from the slides and 100–125 μL of ready to use S100 (Roche Ventana# 790-2914) antibody was applied, and the slides were then placed in a humidity chamber for 1h at room temperature. After the incubation period, the slides were thoroughly rinsed with TBS and then with TBST. The buffer was removed and Mach 2 Mouse HRP-Polymer (MHRP520) was applied, and slides were returned to the humidity chamber for 30 min at room temperature. After the incubation period, the slides were thoroughly rinsed with TBS and then with TBST. The buffer was removed and Deep Space Black Chromogen (BRI401) was applied to slides for 3–5 min. Slides were thoroughly rinsed with distilled water and transferred to TBST. The block was removed from the slides and 100–125 μL of either, HES-1 antibody (Cell signaling #11988, 1:200 dilution), NOTCH1 antibody (Millipore Sigma 07-1231, 1:100 dilution), MAP2 antibody (Thermo Fisher #PA5-24589, 1:50 dilution), NOTCH2 (Sigma #HPA048743, 1:1000 dilution) or Ki-67 antibody (Abcam #ab16667, 1:100 dilution) (for NICD slide) diluted with Da Vinci Green Diluent (PD900) was applied, and slides were placed in a humidity chamber overnight at 4 °C. After the incubation period, the slides were thoroughly rinsed with TBS and then with TBST. After the buffer was removed, Mach 2 Rabbit AP Polymer (RALP525) was applied, and the slides were returned to the humidity chamber for 30 min at room temperature. After the incubation period, the slides were thoroughly rinsed with TBS and then with TBST. The buffer was removed and Warp Red Chromogen (WR806) was applied to the slides for 10 min. The slides were thoroughly rinsed with distilled water and counterstained with Harris Modified Hematoxylin-Fisher (#SH26-500D; diluted to 1:5 with distilled H_2_O) and rinsed with running tap water and then with distilled H2O before placing them in the oven at 58 °C for 30 min and then put through graded alcohols and cleared in xylene. Stained sections on slides were prepared for storage by sealing them with coverslips using Cytoseal™ XYL (Thermo Scientific, Waltham, MA, USA).

## 3. Results

### 3.1. Conditional Knockout of Notch1 or Notch2 in Mouse Melanocytes Accelerates Melanoma Tumor Development and Growth

We crossed the Tyr-CreERT2; *Braf^V600E/+^*;*Pten*^-/-^ (BP)*;* mice with either *Notch1*^tm2Rko^/GridJ mice carrying floxed *Notch1* or *Notch2^tm3^*Grid/J mice carrying floxed *Notch2* to generate BPN1 and BPN2 mice. We also used *Braf^V600E/+^; Pten^+/+^* (B) mice with wildtype Pten. In BP mice, a single topical application of 4-hydroxy tamoxifen (4OHT) is sufficient to induce melanoma tumors on the skin of BP mice, whereas only nevi develop from similar application on the skin of B mice [10]. Flank skin was shaved and 2 µL of 5 mM 4OHT was applied one time at two different spots on each shaved flank. The animals were monitored for tumor formation at the sites of 4OHT application. The tumors were measured using a digital caliper, and animals were monitored until at least one of the four tumors on each mouse reached a measurement of 10 mm in any direction. The mice were then euthanized, and the tumors were excised for further analysis. 

### 3.2. Notch1 or Notch2 Loss Cannot Substitute for Pten Loss or Cooperate with BRAFV600E in Melanomagenesis

**Figure 1:**
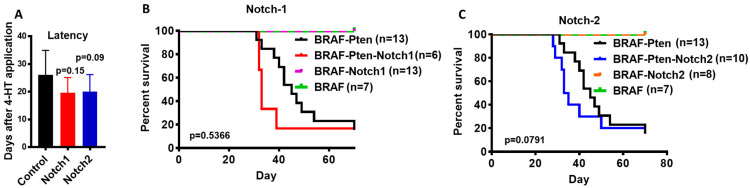
Role of Notch1 and Notch2 in melanomagenesis. (**A**) Tumor latency determined by the time between 4OHT application and the first appearance of a palpable tumor [Control BP mice (*n* = 14), Notch1 knockout mice (*n* = 5), Notch2 knockout mice (*n* = 9)]. B and C. Kaplan–Meier Survival analysis. Each mouse was monitored until at least one tumor on any flank reached 10mm in any direction. In (**B**), BRAF-Pten mice (in black) served as controls. BRAF-Pten-Notch1 mice (in red); (BRAF) mice (in green) are controls for (BRAF-Notch1) mice (in purple). In (**C**), (BRAF-Pten) mice (in black) are controls for (BRAF-Pten-Notch2) mice (in blue); (BRAF) mice (in green) are controls for (BRAF-Notch2) mice in orange.

We first tested whether deletion of *Notch1* (BN1 mice) or *Notch2* (BN2) can substitute loss of *Pten* or cooperate with BRAFV600E in melanomagenesis. We shaved the flanks of B, BN1 and BN2 mice and applied 4OHT. As previously reported [22], the application of 4OHT did not induce melanoma tumors in B mice with an intact Pten tumor suppressor. Similarly, none of the mice in BN1 and BN2 groups developed tumors until the 7-week endpoint. These data show that the loss of neither *Notch1* or *Notch2* is sufficient to induce melanoma by BRAFV600E (Figure 1B,C), and that neither Notch1 nor Notch2 can substitute for the tumor suppressor function of Pten.

We used BP, BPN1 and BPN2 mice to test the effect of loss of *Notch1* or *Notch2* on melanomagenesis in the context of activated BRAFV600E and the loss of Pten. As shown in Figure 1A, the loss of *Notch1* or *Notch2* in melanocytes appeared to accelerate tumor formation and growth in BP mice, and lead to shorter survival compared to control mice (Figure 1B,C). However, the difference in survival was not significant (*p* = 0.54 for Notch1, and *p* = 0.079 for Notch2). Our data, however, suggest that the absence of *Notch1* or *Notch2* tends to accelerate tumor development in BP mice.

### 3.3. Immunohistochemical Analysis of Melanoma Tumors

**Figure 2:**
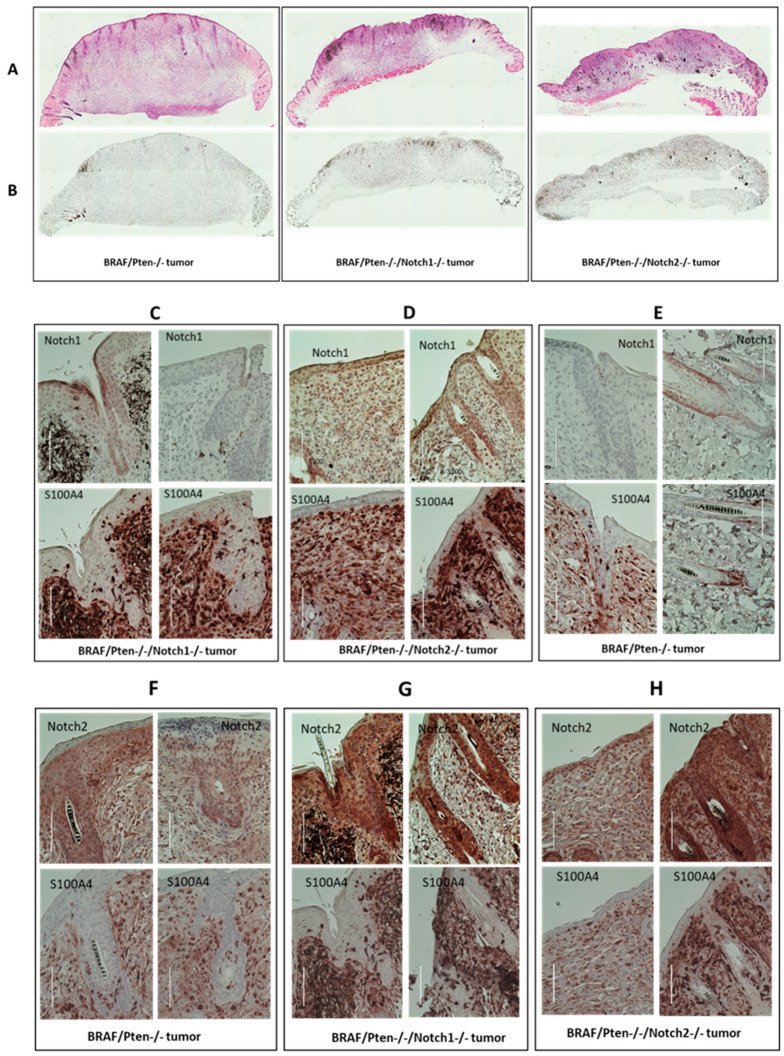
Histological and immunohistochemical analyses for tumors from BP (BrafCa/+/Pten-/-), BPN1(BrafCa/+/Pten-/-/Notch1-/-) and BPN2 (BrafCa/+/Pten-/-/Notch2-/-) mice. (**A**) H&E staining for the three different types of tumors. (**B**) Immunohistochemical staining with Ki67 antibody. (**C**–**E**) Immunohistochemical staining with Notch1 and S100A4 antibodies. (**F**–**H**). Immunohistochemical staining with Notch2 and S100A4 antibodies. Scale bar equals 100 µm.

We performed histological (hematoxylin and eosin, H&E, staining) and immunohisto-chemical (IHC) analyses of control (BP) and BPN1 and BPN2 tumors to define their histological characteristics, confirm the loss of Notch1 and Notch2 expression, and deter-mine the proliferative status (as assessed by Dr. Jack Longley, a dermatopathologist). All tumors showed similar histology including cells exhibiting Schwannian differentiation and neuroid bodies (Figure 2A). IHC staining for the proliferation marker Ki67 showed more proliferative cells in Notch1 knockout and Notch2 knockout tumors compared to the control (Figure 2B). Tumors from the Notch1 knockout group had no detectable Notch1 staining (Figure 2C), whereas tumors from the Notch2 knockout group were positive for Notch1 (Figure 2D). Paradoxically, tumors from the control group also show no Notch1 expression (Figure 2E). It is possible that BP mouse melanocytes that form melanoma do not express Notch1, and that the Notch1 expression seen in tumors from the Notch2 knockout group is a result of Notch2 knockout, i.e., compensating for the loss of Notch2 expression. Alternatively, tumors in the BP mice develop from Notch1-negative cells or have downregulated Notch1 during tumorigenesis. On the other hand, Figure 2F–H show tumors from all three different types including tumors from the Notch2 knockout mice group stained for Notch2. These data suggest a dynamic interaction between oncogenic BRAF signaling and Notch expression.

### 3.4. Evaluation of Notch Signaling in Human Primary Melanoma Tumors

**Figure 3:**
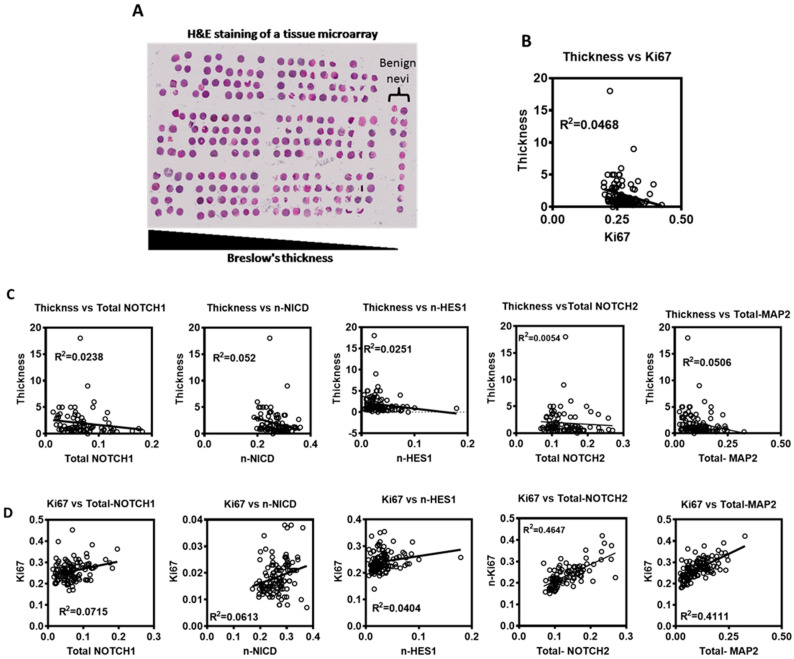
Notch signaling expression in human primary melanoma TMA. (**A**) Representative hematoxylin and eosin staining (H&E) of the used TMA slides. (**B**–**D**) A linear regression analysis was used to assess the relationship between different markers and parameters in human primary melanoma tumors: (**B**) Tumor thickness vs. Ki67. (**C**). Tumor thickness vs. (total NOTCH1, nuclear NICD, nuclear HES1, total NOTCH2, or total MAP2). (**D**). Ki67 vs. (total NOTCH1, nuclear NICD, nuclear HES1, total NOTCH2, or total MAP2).

To evaluate expression of Notch signaling proteins and their significance in human melanoma, we employed a tissue microarray (TMA) built in-house from the archival collection of paraffin embedded primary melanoma tumor specimens from patients diagnosed with primary cutaneous melanoma. This TMA consists of >200 tumor specimens with complete clinical follow up data including recurrence and survival (Figure 3A). BRAFV600E mutant tumors constitute >60% of tumors on this TMA, indicating that this cohort of melanomas is a representative cohort of melanoma patients. Serial sections of the TMA were stained for (NOTCH1, Ki67 and S100A4), (NICD, Ki67 and S100A4), (HES1, Ki67 and S100A4), (NOTCH2, Ki67 and S100A4) or (MAP2, Ki67 and S100A4). Using the Vectra slide scanner and InForm software, automated multi-spectral image acquisition and analyses was done. Total cellular (cytoplasmic and nuclear) and nuclear expression of NOTCH1, NICD, HES1, NOTCH2, MAP2, and Ki67 proteins was quantitated in S100A4-positive cells. 

Analysis of these markers showed a weak to moderate correlation between widely used clinical prognostic parameters. We found a weak negative correlation between Breslow thickness and the proliferative status (Ki67) (Pearson Correlation, r^2^ = 0.0468) (Figure 3B). Also, a weak negative correlation was found between total (nuclear + cytoplasmic) NOTCH1 protein expression, nuclear NICD, nuclear HES1 (an effector of Notch signaling and a transcription factor/repressor), total NOTCH2 and total MAP2 (neuronal differentiation marker) and Breslow thickness (Pearson Correlation, r^2^ = 0.02382, 0.05243, 0.02512, 0.0054, 0.0506, respectively) (Figure 3C). A weak to moderate positive correlation was found between Ki67 and each of total NOTCH1, nuclear NICD, nuclear HES1, total NOTCH2 and total MAP2 (Pearson Correlation, r^2^ = 0.07154, 0.06132, 0.04047, 0.4647, 0.4111, respectively) (Figure 3D). These data suggest that Notch signaling is involved in the early proliferative stage of primary melanoma. 

### 3.5. Prognostic Value of Notch Signaling in Melanoma

To test the prognostic significance of Notch signaling proteins in primary melanoma, we first stratified tumors as high and low (top and bottom quartiles) expressors according to the level of total or nuclear expression based on optical density (OD) values for each marker protein. A Kaplan-Meier survival analysis was performed to test the relationship between the marker protein expression and five year-disease free survival (DFS) after the initial excision of the primary melanoma tumor.

For NOTCH1 expression, the cut-off values were OD ≤ 0.045 (for low expression) OD ≥ 0.086 (for high expression). Our data show no significant difference in DFS between patients with high and low total NOTCH1 expression (Log-rank Mantel-Cox test *p*-value= 0.4755 or 0.11, respectively). 

**Figure 4:**
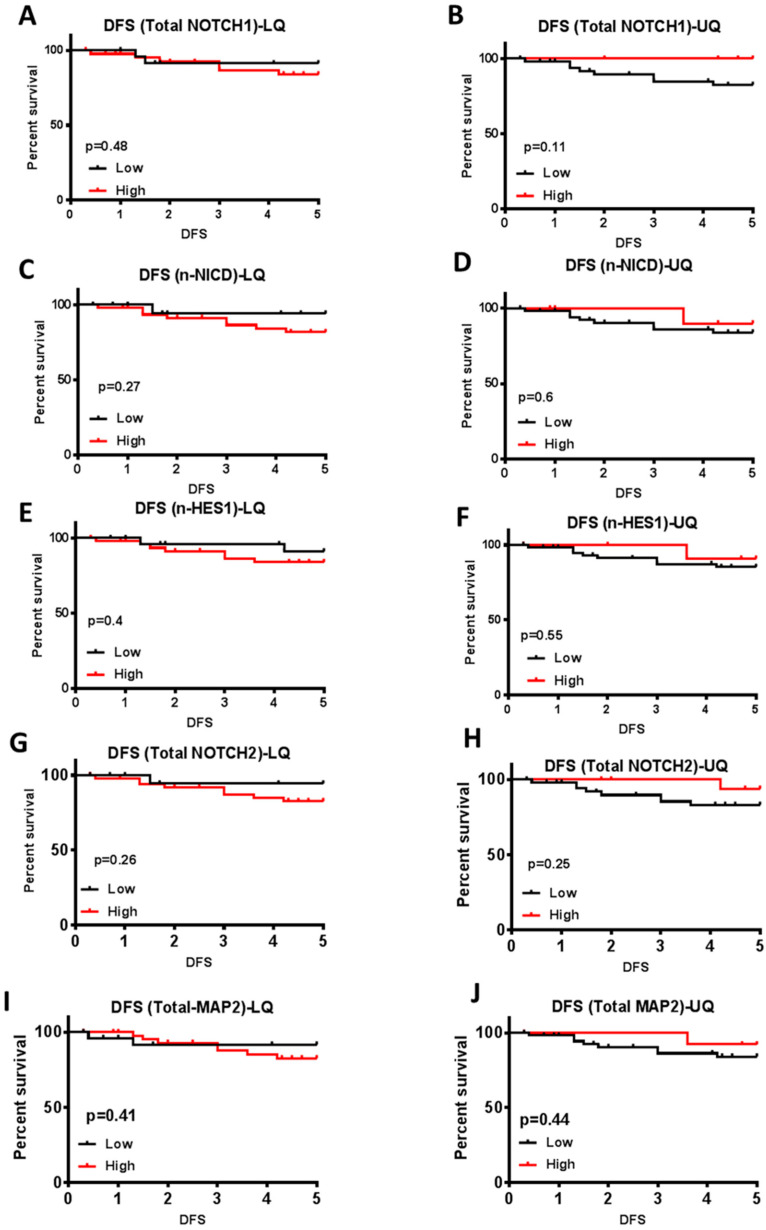
Kaplan–Meier Survival analysis for five year-disease free survival of patients diagnosed with primary melanoma tumors. (**A**) Low total NOTCH1 = optical density of 0.045 or less (lower quartile). (**B**) Low total NOTCH1 = optical density of 0.086 or less (upper quartile). (**C**) Low NICD = optical density of 0.235 or less (lower quartile). (**D**) Low NICD= optical density of 0.298 or less (upper quartile). (**E**) Low nuclear HES1= optical density of 0.017 or less (lower quartile). (**F**) Low nuclear HES1 = optical density of 0.038 or less (upper quartile). (**G**). Low total NOTCH2 = optical density of 0.0985 or less (lower quartile). (**H**) Low total NOTCH2 = optical density of 0.1655 or less (upper quartile). (**I**) Low nuclear MAP2 = optical density of 0.066 or less (lower quartile). (**J**) Low nuclear MAP2 = optical density of 0.172 or less (upper quartile).

Patients with tumors that had low total NOTCH1 expression showed nearly half the recurrence rate compared to patients with high total NOTCH1 [Hazard Ratio (HR) = 0.564, 95% CI = 0.1345 to 2.368] (Figure 4A). On the other hand, when using the upper quartile cutoff estimates that patients with low total NOTCH1 had almost four times higher recurrence rate than patients with high total NOTCH1 (HR = 3.794, 95% CI =0.7467 to 19.28] (Figure 4B). These data are consistent with NOTCH1 expression being a feature of less aggressive melanoma but fail to demonstrate a significant association. 

The effect of NOTCH2 expression on DFS and the probability of metastasis also showed a similar pattern as NOTCH1 [OD ≤ 0.0985 vs. ≥0.1655 for low and high expression: Log-rank *p*-value= 0.26 or 0.25, respectively, and HR= 0.3202, 95% CI = 0.07482 to 1.37 for lower quartile cutoff (Figure 4G), and HR = 3.162, 95% CI= 0.7418 to 13.48 for upper quartile cutoff (Figure 4H)]. 

Similarly, nuclear NICD expression (OD ≤ 0.235 vs. ≥ 0.298 for low and high expression) also showed no significant prognostic value for DFS (Log-rank Mantel-Cox test *p*-value = 0.27, or 0.5932, respectively). Patients with tumors exhibiting low nuclear NICD expression had nearly one third chance of recurrence compared to those with high nuclear NICD expression (HR = 0.3315, 95% CI = 0.07658 to 1.434) (Figure 4C). When the upper quartile was used as the cutoff, patients with low nuclear NICD expression had approximately a two times higher recurrence rate compared to patients with high nuclear NICD expression (HR = 1.745, 95% CI = 0.3176 to 9.591) (Figure 4D), suggesting that the higher expression of active NOTCH1 may delay or inhibit metastatic recurrence in melanoma patients. 

The amount of Notch signaling effector HES1 (OD ≤ 0.017 vs. ≥ 0.038 for low vs. high) also showed no significant effect on DFS (Log-rank Mantel-Cox test *p*-value = 0.4023 or 0.5457, respectively). However, patients with low nuclear HES1 had almost half the DFS rate compared to patients with high nuclear HES1 (HR = 0.5183, 95% CI = 0.1324 to 2.03) (Figure 4E), and when the upper quartile was used as the cutoff, patients with low HES1 expression had nearly twice the probability of recurrence compared to patients with high HES1 expression (HR = 1.874, 95% CI = 0.3542 to 9.916) (Figure 4F). These data show that nuclear HES1 has a similar effect as total NOTCH1 and NICD1 on melanoma metastasis. 

Interestingly, the neuronal differential marker MAP2 also showed a correlation to DFS and a probability of metastatic recurrence as NOTCH proteins [OD ≤ 0.063 vs. ≥ 0.141 for low vs. high expression; Log-rank test *p*-value = 0.41 and 0.44, respectively; HR = 0.525, 95% CI = 0.1337 to 2.061 with lower quartile (Figure 4J), and HR = 2.223, 95% CI = 0.456 to 10.83 for upper quartile (Figure 4I)]. These data suggest that patients with tumors that express higher MAP2 expression tend to have a lower rate of metastatic recurrence and thus a more favorable outcome. 

## 4. Discussion

In this study, we investigated the role of Notch signaling in melanocyte transformation in the context of oncogenic BRAF and the activation of PI3 kinase signaling [20]. There is some evidence that signaling pathways involved in melanomagenesis modulates Notch signaling. For example, the inflammatory response caused by PI3K/Akt signaling is known to promote Notch-induced cancer development [23]. 

### 4.1. Notch Proteins as Melanoma Tumor Suppressors

An important finding from our in vivo study is that although neither Notch1 nor Notch2 acts as a tumor suppressor to fully substitute Pten in BRFAV600E induced melanomagenesis, the loss/decrease of Notch proteins appeared to accelerate tumor formation and promote tumor growth. These data point to a role for Notch signaling in dampening the oncogenic effect of BRAF and the tumor suppressor function of Pten. In this context it is noteworthy that a recent report implicated NOTCH4 as a tumor suppressor in melanoma. The constitutive expression of NICD4 in melanoma cells was shown to cause a phenotypic switch from a mesenchymal-like parental phenotype to an epithelial-like phenotype (MET), delayed tumor formation, and significantly reduced tumor volume in vivo [5]. 

### 4.2. Role of Notch1 in Melanoma Tumorigenesis and Progression

On the other hand, using mouse xenograft models, several studies have implicated an oncogenic role for Notch in melanoma [11,24]. In one such study, the constitutive expression NICD1, the activated form of Notch1, was shown to induce the aggressive growth of vertical growth phase (VGP) primary melanoma cell lines in vivo with metastatic activity [4]. Notch signaling inhibition was shown to suppress melanoma cell growth in vivo, whereas Notch activation seems to promote the progression of primary melanoma cells to metastasize to the lungs [9]. Blocking Notch signaling caused melanoma tumors to shrink in vivo [8]. In another xenograft model, NOTCH1 inhibition was shown to cause a reduction in cell growth, increased cell death, and delayed tumor growth [10]. Notch inhibition was shown to reduce the growth of human primary melanoma xenografts in mice and inhibit their serial xenotransplantation. Notch inhibition also reduced the tumor volume of metastatic melanoma cell line xenografts in mice [11]. Although these studies are useful, unlike the genetic model we employed, xenograft models and pharmacological inhibition studies have inherent limitations such as tumor growth in an immune-deficient environment and the in vivo specificity of Notch inhibitors. These limitations preclude a definitive assignment of an oncogenic or tumor suppressor role for Notch.

### 4.3. Prognostic Relevance Notch Signaling in Human Cutaneous Melanoma

In this study, we also report a first comprehensive quantitative immunochemical analysis of expression of Notch signaling proteins and their prognostic significance in human primary melanoma. Our study revealed that a weak to moderate positive correlation was detected between each of NOTCH1, NICD, HES1 and NOTCH2 and the proliferative status (Ki67 levels). A weak negative correlation was detected between each of these five markers NOTCH1, NICD, HES1, NOTCH2, and Ki67 and Breslow thickness, suggesting that Notch signaling plays a suppressive role in the initial proliferative stages. Although the expression of HES1 and DLL3, which are components of the Notch signaling pathway, was shown to correlate with shorter post recurrence survival in patients with metastatic melanoma, to the best of our knowledge, no other studies have investigated the prognostic value of Notch in primary melanomas [11]. 

### 4.4. Notch Signaling and Neuronal Differentiation Markers in Human Cutaneous Melanoma

The expression of MAP2, which is negatively regulated by Notch signaling, was shown previously by our laboratory, in a small cohort of primary melanomas, to predict a better outcome for primary melanoma patients. Patients with longer metastatic DFS had high MAP2 expression compared to those with shorter DFS and weak or no MAP2 expression [25]. Using primary tumors from a larger cohort, we showed that MAP2 expression did not significantly predict a longer disease-free survival for primary melanoma patients. This might be due to the different experimental approaches that were used in these two studies. While the previous study categorized tumors on a positive/negative or high/low (non-continuous variable) MAP2 expression basis, in this study we used quantitative immunohistochemistry to categorize the tumors as high/low MAP2 expression. Nevertheless, although the *p*-value did not show significance, patients with high MAP2 still showed a tendency toward longer DFS compared to patients with low MAP2. 

## 5. Conclusions

In this study, we investigated (a) the role of Notch1 and Notch2 in melanomagenesis in vivo in a mouse model, and (b) the prognostic significance of Notch signaling proteins in human cutaneous primary melanoma. Our data show that loss of Notch 1 or Notch2 cannot substitute for the tumor suppressor functions of Pten in BRAFV600E-induced melanoma. However, Notch1 and Notch2 can delay tumor development and inhibit their growth once BRAF-induced tumors are initiated in Pten-null mice. In human cutaneous primary melanoma, the expression of Notch signaling proteins also appear to be negatively correlated with melanoma cell proliferation and predict the probability of metastatic recurrence. In conclusion, our studies unravel a subtle role, albeit an inhibitory role, for Notch signaling in cutaneous melanoma. Identifying proteins that mediate this inhibitory action could help design strategies for the prevention of melanoma progression.

## Data Availability

Not applicable.
